# Face touch monitoring using an instrumented wristband using dynamic time warping and k-nearest neighbours

**DOI:** 10.1371/journal.pone.0281778

**Published:** 2023-02-17

**Authors:** Ramin Fathian, Steven Phan, Chester Ho, Hossein Rouhani

**Affiliations:** 1 Department of Mechanical Engineering, University of Alberta, Edmonton, Alberta, Canada; 2 Department of Medicine, University of Alberta, Edmonton, Alberta, Canada; Vellore Institute of Technology: VIT University, INDIA

## Abstract

One of the main factors in controlling infectious diseases such as COVID-19 is to prevent touching preoral and prenasal regions. Face touching is a habitual behaviour that occurs frequently. Studies showed that people touch their faces 23 times per hour on average. A contaminated hand could transmit the infection to the body by a facial touch. Since controlling this spontaneous habit is not easy, this study aimed to develop and validate a technology to detect and monitor face touch using dynamic time warping (DTW) and KNN (k-nearest neighbours) based on a wrist-mounted inertial measurement unit (IMU) in a controlled environment and natural environment trials. For this purpose, eleven volunteers were recruited and their hand motions were recorded in controlled and natural environment trials using a wrist-mounted IMU. Then the sensitivity, precision, and accuracy of our developed technology in detecting the face touch were evaluated. It was observed that the sensitivity, precision, and accuracy of the DTW-KNN classifier were 91%, 97%, and 85% in controlled environment trials and 79%, 92%, and 79% in natural environment trials (daily life). In conclusion, a wrist-mounted IMU, widely available in smartwatches, could detect the face touch with high sensitivity, precision, and accuracy and can be used as an ambulatory system to detect and monitor face touching as a high-risk habit in daily life.

## Introduction

Stopping or slowing the spread of infectious diseases, particularly COVID-19, is a top priority for governments at the national and community levels around the world. Measures and regulations are strictly enforced to control both direct and indirect transmission of infection [1]. Droplets spread by inhalation, and surfaces exposed to the droplets are the primary means of infection transmission [1]. As a result, measures such as detecting and isolating infected individuals, travel restrictions, quarantine, social distancing, and personal hygiene recommendations are implemented. Changing high-risk habitual behaviours is a critical action that leads to stopping or slowing the spread of the COVID-19 infection and breaking the transmission chain during the pre-asymptotic and asymptomatic stages. The perioral and perinasal regions are entry points into the body for the infection [1, 2]. Face touching is a habit that frequently occurs throughout the day and the primary factor in COVID-19 surface-mediated transmission. According to one study, people touch their faces 23 times per hour on average [3]. Since COVID-19 may persist on surfaces for a while, hands can become contaminated easily. Once the hand has been contaminated, a high-risk habit of face touching or unsafe gestures could spread the infection to the mouth, nose, and eye. In another study, it was observed that even in patients suspected of having COVID-19 in the waiting room, the number of face touches was high [4]. The high number of face touches demonstrates the importance of using a personal monitoring system to provide the user with feedback on high-risk activities [4, 5]. This personal monitoring system can be life-saving for individuals working in high-risk environments, such as healthcare workers.

The measurement systems for human motion tracking and gesture recognition can be categorized into two types: (1) stationary motion tracking systems which usually use cameras, including stereo cameras and depth-aware cameras that are with or without markers [6–9]., and (2) wearable sensors including inertial measurement units (IMU), electromyography sensor, magnetometer sensor, and flex/stretchable sensor [9–16]. While both of these measurement systems along with feature selection and classification methods could be used for hand motion recognition, only wearable sensors are practical for gesture recognition during several hours of daily life [17]. In contrast to the stationary motion tracking systems which are cumbersome and limited to a dedicated space, wearable sensors are portable and easy to use and thus have a great potential to be used in gesture recognition and human-computer interface [12, 18–24].

Flex/stretchable and EMG sensors are among the common wearable sensors that have been used to recognize the wrist and finger motion by mapping the skin and muscles to a specific wrist or finger motion [12, 15, 21]. The primary application of these technologies is the human-machine interface and neural prostheses. Another common wearable sensor is IMU. Unlike flex/stretchable and EMG sensors, the IMU is widely available in smartwatches and bands, and is able to unobtrusively record wrist motion without a need for skin preparation and secure and precise sensor attachment. The IMU has shown to have a capability to recognize hand motions that involve arm motion, for applications such as smoking gestures during several hours of daily life [16, 25, 26].

Wearable sensors were also used to detect face touch. Previous research studies proposed methods for face touch detection using an accelerometer, magnetometer, and acoustic-based system and studied the validity of these methods [27–33]. Marullo et al. [27] proposed recurrent neural network (RNN) based methods to detect face touch and provide real-time feedback using accelerometer readouts collected from a smartwatch. The true positive rate and false positive rate were 100% and 3.1%, respectively, for the best method. The experiment was designed to include confounding gestures performed while the participants were sitting and walking. Rojas et al. [28] proposed an acoustic-based system that was able to recognize the face touch using an ear-set. To recognize the face touch, an audio file was played on an ear-set placed on the neck to emit an ultrasound signal that was continuously recorded by the microphone on an ear-set. This way, the distorted ultrasound signal caused by the hand motion was recorded by the ear-set microphone and was then classified using a machine learning model. They obtained 90.0 to 96.0% sensitivity and 86.5 to 98.3% precision in detecting face touches, depending on the user activity. D’Aurizio et al. [29] presented two methods that were able to detect face touch and warn the individuals. The first method relied on three components, a smartwatch placed on the hand, a processing unit that could be a smartwatch or companion smartphone, and a magnetic wearable accessory (like a necklace) that needed to be worn close to the face. In this method, the accelerometer and gyroscope readouts were used to estimate the orientation. Then, the estimated orientation and the magnetometer readout (that could be affected by being close to the magnetic wearable accessory) were used to detect the face touch. This method showed a correctly detected rate and false positive rate of 91.3% and 3.2%. The second method relied on the inertial measurements obtained by the smartwatch. This method showed a correctly detected rate and false positive rate of 92.6% and 38.1%. Both methods were only tested in controlled environment trials while the participants were asked to perform face touch as well as confounding gestures (like eating with a spoon, drinking with a mug, hair-combing, and putting on a t-shirt). A DTW classifier was proposed by Chen et al. [30] that was using accelerometer readout to detect face touch with 99% and 85% accuracy in user-dependent and user-independent. In another studies, Michelin et al. [31] and Alesmaeil et al. [32] investigated the ability of IMU and Convolutional Neural Networks (CNN) to detect face touch. The method proposed by Michelin et al. was validated on the data collected from 40 participants during sitting, standing, and walking trials and obtained an accuracy of 95.7%. Their experimental setup was composed of an IMU, a processing unit (laptop), and a cable to connect the IMU to the laptop. Alesmaeil et al. [32] validated their CNN-based method on 5 participants and obtained an accuracy of 97%. In addition, an IMU-based Random Forest algorithm was developed with an accuracy of 88.4% by Roy et al. [34]. They included confounding gestures such as scratching the head, picking up an item from the floor, and reaching to a shelf, as well as sitting, standing, and walking. In this study, 10 participants were instructed to perform face touch and confounding gestures during sitting, standing, and walking trials. Most of the methods available in the literature [27–34] showed high accuracy in detecting face touch during their designed experimental procedure.

Based on IMU’s technical and practical capacities, we hypothesized that the recordings of a wrist-worn IMU representing the wrist motion could be used to recognize the face touch gesture with high sensitivity and precision. High sensitivity is required to minimize the risk of unrecognized face touching, while high precision is needed to minimize the number of false alarms that help increasing the technology’s compliance during daily life. Therefore, the objectives of this study are to (1) develop a technology to detect and monitor the face touch using a wrist-worn IMU based on a machine learning technique, (2) validate the developed technology in controlled environment trials and (3) investigate the accuracy of our developed technology under natural environment (daily life).

## Methods

**Fig 1:**
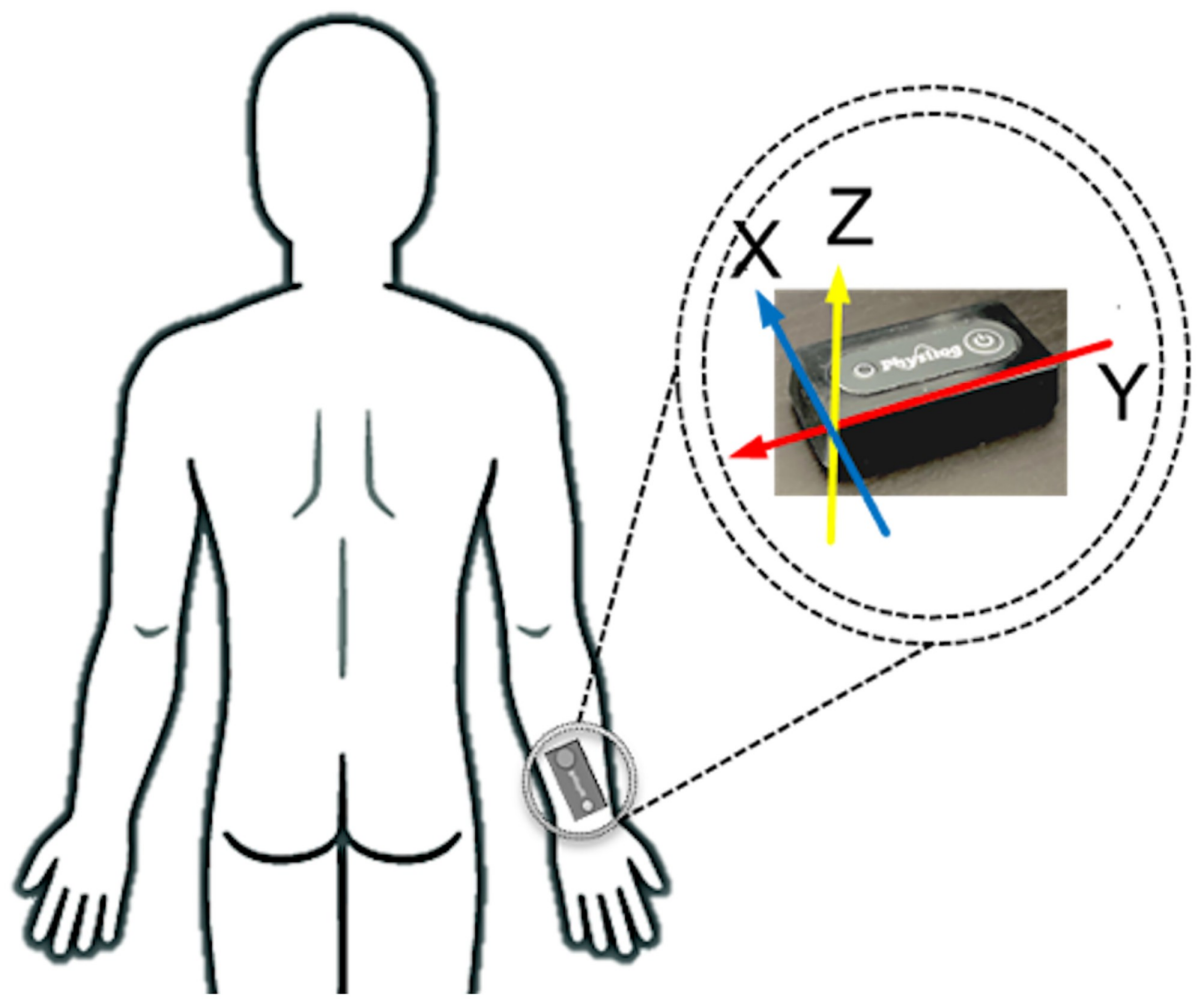
IMU placement on the wrist and the IMU coordinate system.

To have a minimal effect on the pattern of hand motion and daily life activities, a stand-alone IMU module (Physilog 5, Gait Up, Switzerland) that included an accelerometer and gyroscope was used to record the three-dimensional (3D) acceleration and angular velocity at a sampling frequency of 128 Hz. The IMU was mounted on the dominant wrist of the participants using a Velcro strap (Fig 1), and the participants were able to switch it on and off by pressing the start/stop button.

### A. Experimental procedure

Eleven volunteers (six males and five females, age: 26 ± 3 years, body height: 170 ± 10 cm, body mass: 69 ± 14 kg) participated in the study. The experiment procedure was approved by the Research Ethics Board Committee of the University of Alberta (Pro00102526) and all methods were performed in accordance with the approved experiment procedure. Participants were asked to read and sign the written informed consent form prior to participating in the experiment. The experiment included two parts: controlled environment and natural environment trials.

During the controlled environment trials, the participants were asked to perform six different scenarios (1. Face touching, 2. Handwashing, 3. Eating with various utensils, 4. Drinking, 5. Scratching head, and 6. Reaching and picking an object) each for five times. Each scenario contained at least five repetitions of a single gesture leading to at least 25 repetitions per participant for every single gesture. They performed these motions with their dominant hand while the IMU recorded their wrist motion (Fig 1). In addition, at the beginning and end of each scenario, participants were requested to start and stop the IMU recording by pressing the start/stop button. During each scenario, participants were instructed to have 10 seconds of rest position at the beginning and end of the trials.

In addition to controlled environment trials, natural environment trials were included to investigate the ability of our developed algorithms in detecting gestures in daily life activities. In natural environment trials, participants were asked to follow their daily life activities while occasionally performing the abovementioned gestures (face touching, handwashing, eating drinking, scratching head, and reaching and picking an object) and log their activities for 20 minutes. As a result, 20 minutes of the participants’ routine daily life activity which contained a known number of face touches were recorded while the IMU was mounted on their dominant wrist. Similar to the controlled environment trials, each 20-minute trial was also started and ended with 10 seconds of in the rest position recording.

Participants logged the approximate instants in which face touches were performed in a log sheet which was then used to label the IMU recording. Additionally, a repetition of face touching gesture was judiciously selected from the previously recorded data as a template which was then used for feature extraction.

### B. Data analysis

#### Pre-processing of the IMU reading

**Fig 2:**
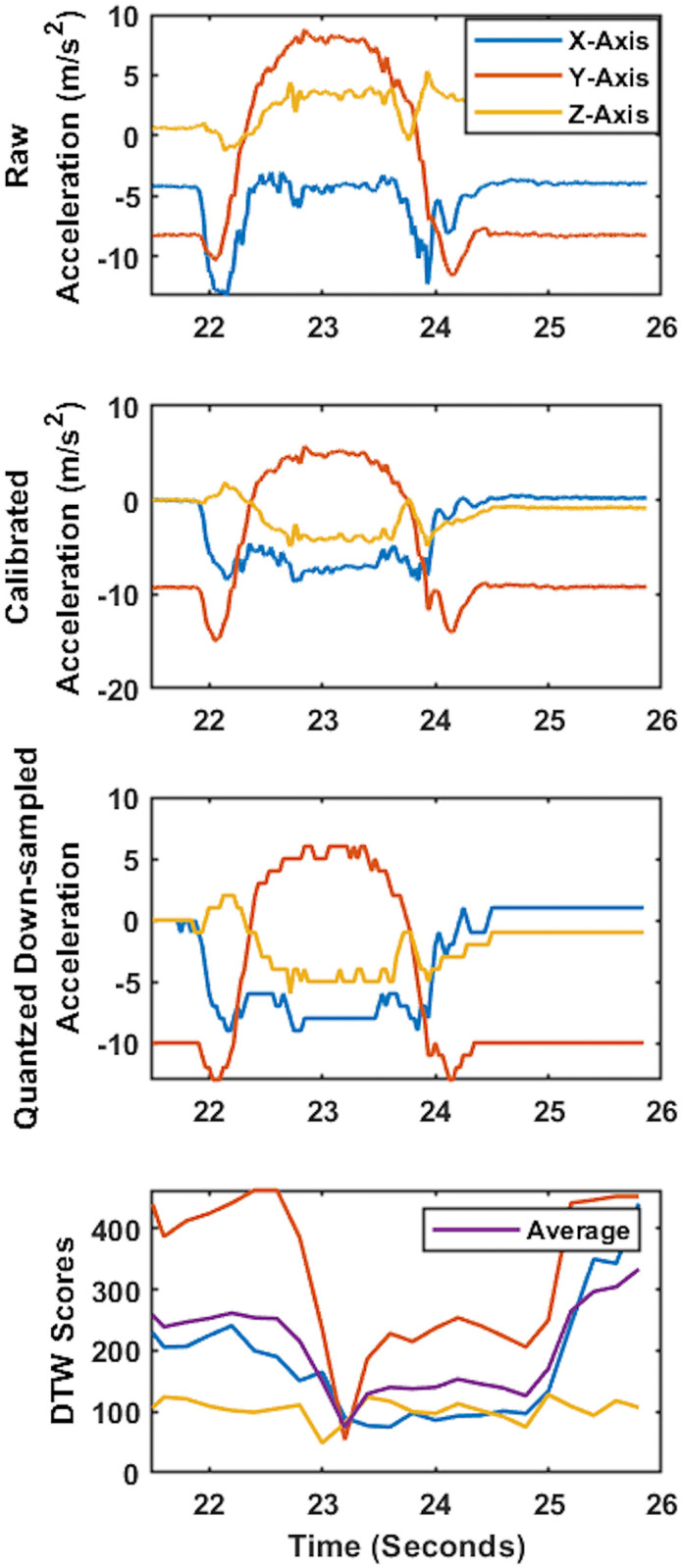
Sample 3D acceleration time-series and associated DTW scores. a) Raw 3D acceleration time-series during a face touch, b) acceleration time-series after vertical alignment, c) acceleration time-series after down-sampling and quantization, and d) the DTW scores calculated comparing the pattern of quantized down-sampled acceleration time-series to template time-series (lower score represents more similarity). Considering that the face touch occurred from 22 to 25 sec, it could be observed that in this sample DTW scores decreased while the face touch happened (23 to 24 sec).

**Fig 3:**
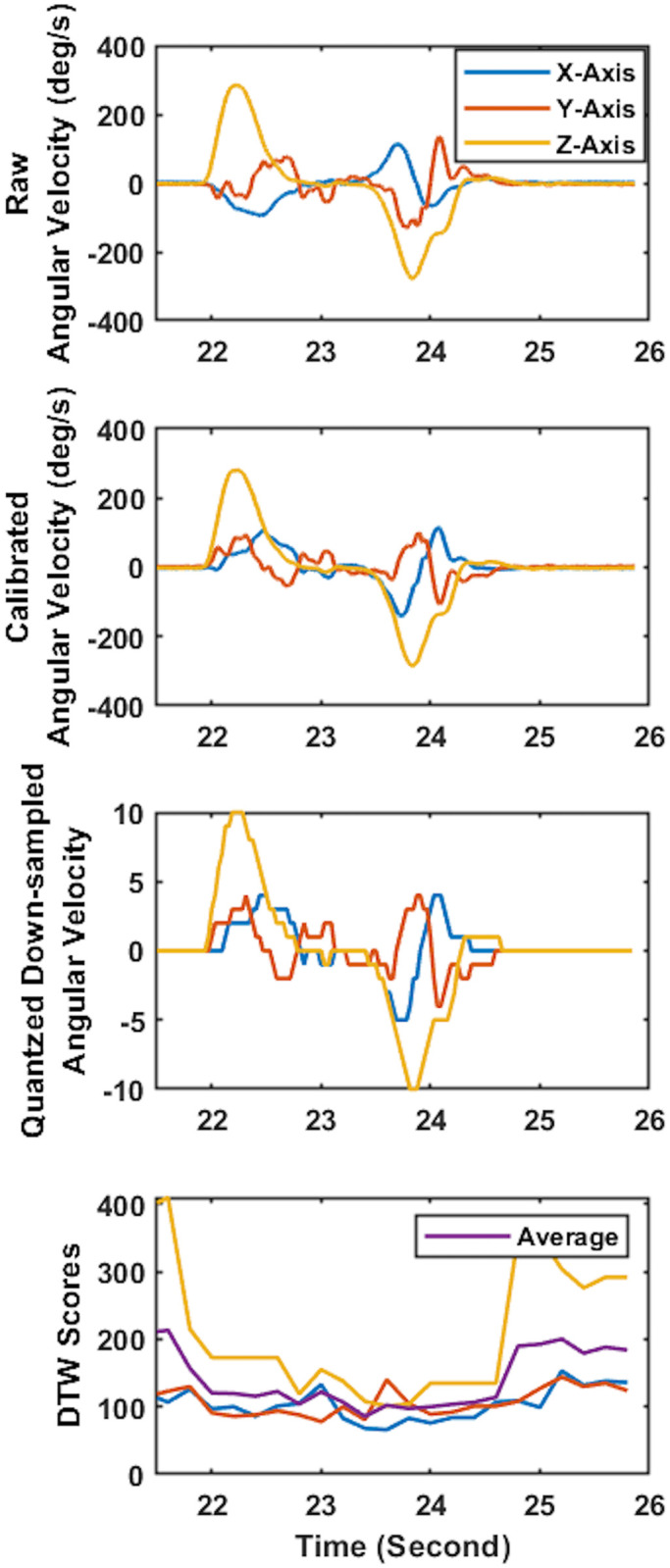
Sample 3D angular velocity time-series and associated DTW scores. a) Raw 3D angular velocity time-series during a face touch, b) angular velocity time-series after vertical alignment, c) angular velocity time-series after down-sampling and quantization, and d) the DTW scores calculated comparing the pattern of quantized down-sampled angular velocity time-series to template time-series.

After each test session, the IMU recordings that contained the acceleration and angular velocity signal were used as the model’s input data. To remove the noise introduced by skin artifacts, the acceleration and angular velocity data were filtered using zero-delay 6^th^-order low-pass Butterworth filter with a cut-off frequency of 30Hz. Then, the IMU readouts were transformed from the IMU’s coordinate system into a vertically aligned coordinate system (Figs 2a and 2b, 3a and 3b) to minimize the effect of IMU placement on the wrist on the recordings and improve consistency in recordings among participants [22, 23, 35]. For this purpose, the accelerometer readout obtained from the 10-second quiet standing at the beginning of the first trial was used to find the rotation matrix transforming the acceleration and angular velocity from IMU’s coordinate system to the vertically aligned coordinate system (Figs 2a and 2b, 3a and 3b).

#### Data-labeling

After pre-processing the IMU recordings, all the data collected during controlled environment and natural environment trials were manually labelled. In the natural environment trials, a self-reporting log sheet was used for labelling. The log sheet only contained the approximate face touch instants. In order to reduce the error and bias in labelling the data, the resultant vector of angular velocity obtained by the IMU was calculated and the period in which face touch happened was defined as the instants in which the resultant angular velocity was minimum. Then the accompanying maximum peaks were considered as the start and end time for the face touch. As a result, a label vector that included three categories was generated (1 represented face touch, 2 represented confounding gestures, and 0 represented other).

#### Feature extraction using Dynamic Time Warping (DTW)

Feature extraction was performed on the 3D acceleration and angular velocity recordings obtained by IMU that represented the hand motion during the trials. The similarity of the template to the 3D acceleration and angular velocity recordings was obtained using DTW. A repetition of face touch gesture was judiciously selected from the previously recorded data as a template. The template consisted of acceleration and angular velocity recordings. In order to calculate the similarity score between the template and the recorded acceleration and angular velocity, we constructed a cost matrix. Then, the total distance with the least cost was defined as the DTW score [36]. DTW was used to calculate the similarity since the scores obtained from the DTW were not sensitive to global and local scaling and shifting in the time dimension. The DTW scores were calculated in a 3-second sliding window with a step size of 0.2 second (Figs 2d and 3d). To calculate the DTW scores, the FastDTW technique presented by Salvador et al. was used [37]. The recorded data was down-sampled to 32 Hz and quantized (converted into discrete values) to reduce the computational cost [25, 38] and the effect of floating points in DTW scores (Figs 2c and 3c) [25, 38]. Then, we constructed an 8-dimensional feature space using the calculated DTW scores composed of three feature vectors for acceleration (Fig 2d), three feature vectors for the angular velocity (Fig 2d), and two feature vectors representing the average of the three feature vectors based on acceleration and the average of the three angular velocities.

#### Classification using K-Nearest Neighbours (KNN)

We chose the KNN classifier which is a non-parametric classification method to classify the data and detect face touches. First, the feature space containing the feature vector (DTW scores) and class labels was created [39] using the training set. Then the class labels of the testing set were classified by major voting between *k* nearest neighbours in feature space. The major voting classification was performed for the k equal to 3 to 21. For the purpose of training, the feature vectors among the controlled environment and natural environment trials were randomized separately and then split into 70% and 30% to form the training and test set, respectively. A total number of 82,822 instants that included 1,562 instants of face touching and 38,523 instants of the confounding gestures existed in controlled environment trials datasets, and a total number of 67,190 instants that included 761 instants of face touch existed in natural environment trials datasets.

#### Validation of DTW-KNN model

**Fig 4:**
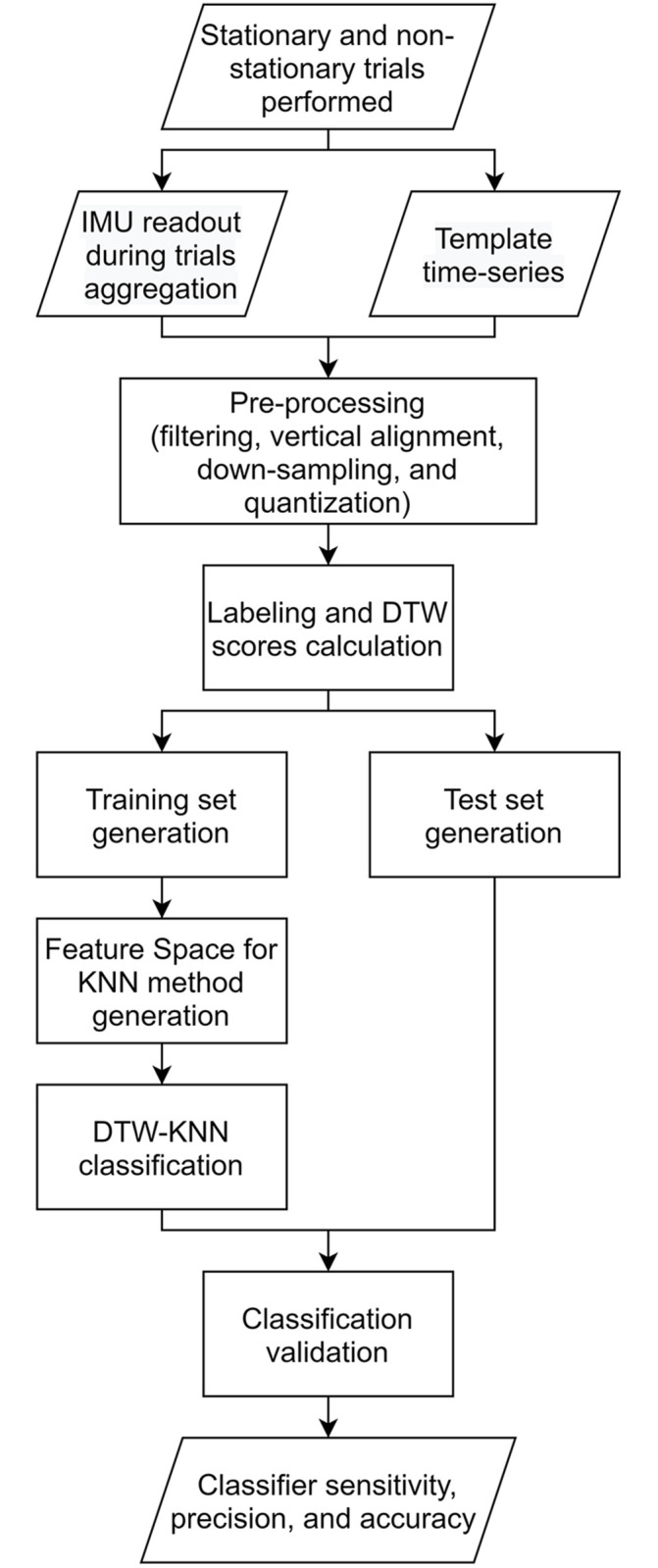
Flowchart representing the data collection, pre-processing, data labeling, feature extraction, classification, and validation.

The classification was performed for each instant in the test set using the KNN model developed based on the training set. Then, for each *k* (number of neighbours) in the range of 3 to 21, each instant in the test set was classified using the KNN model and compared to the true label vector (Fig 4). Note that the label vector defined the entire period of the face touch according to data labeling section. The focus of this study was on estimating the number of face touches and the approximate time of occurrence. Therefore, the periods that were predicted as face touch and had an overlap with the true face touch were considered as correct classification. As a result, there were cases in which that the duration of the periods predicted as face touch were not exactly matching with the true face touches but were still considered as correct classification. In the next step, we calculated the condition positive (RP), condition negative (RN), true positive (TP), true negative (TN), false positive (FP), and false negative (FN), as well as sensitivity, precision, accuracy, false positive rate (FPR), and false discovery rate (FDR), over the entire test set, for each value of *k*. In addition, we evaluated the performance of the model using Leave-One-Subject-Out Cross-Validation to investigate the effect of subject bias in our model. Then, the average accuracy, sensitivity, and precision obtained from the KNN method (*k* from 3 to 21) for 11 folds were calculated.

## Results

The sensitivity, precision, and accuracy for the controlled environment trials were in the range of 81% to 95%, 97% to 100%, and 85% to 93%, respectively, for *k* in the range of 3 to 21 (Table 1a). The sensitivity, precision, and accuracy for the natural environment trials were in the range of 48% to 79%, 47% to 100%, and 48% to 79%, respectively, for *k* in the range of 3 to 21 (Table 1b). In the controlled environment trials, the false positive rate and false discovery rate were in the range of 0% to 1% and 0% to 3%, respectively. The FPR and FDR were not calculated for natural environment trials since not all the gestures (other than face touches) were labelled.

**Table 1:** The number of the condition positive (RP), condition negative (RN), true positive (TP), true negative (TN), false positive (FP), and false negative (FN), as well as sensitivity, accuracy, precision, false positive rate (FPR) and false discovery rate (FDR), calculated for the controlled environment (A) and natural environment trials (B).

(A)										
K	RP	TP	FP	TN	FN	Sensitivity (%)	Precision (%)	Accuracy (%)	FPR (%)	FDR (%)
3	124	113	4	343	11	91	97	85	1	3
5	124	114	2	361	10	92	98	89	1	2
7	124	118	1	376	6	95	99	92	0	1
9	124	112	0	382	12	90	100	92	0	0
11	124	110	0	386	14	89	100	93	0	0
13	124	103	0	379	21	83	100	90	0	0
15	124	100	0	385	24	81	100	90	0	0
17	124	103	0	384	21	83	100	91	0	0
19	124	101	1	377	23	81	99	89	0	1
21	124	106	1	380	18	85	99	91	0	1
(B)										
K	RP	TP	FP	FN	Sensitivity (%)		Precision (%)		Accuracy (%)	
3	29	23	2	6	79		92		79	
5	29	23	3	6	79		88		79	
7	29	19	1	10	66		95		66	
9	29	20	1	9	69		95		69	
11	29	19	0	10	66		100		66	
13	29	15	17	14	52		47		52	
15	29	16	1	13	55		94		55	
17	29	16	0	13	55		100		55	
19	29	15	0	14	52		100		52	
21	29	14	10	15	48		58		48	

The accuracy, sensitivity, and precision values obtained from Leave-One-Subject-Out Cross-Validation using the KNN model were calculated for each *k* in the range of 3 to 21 during the controlled environment and natural environment trials. Then the sensitivity, precision, and accuracy were calculated and tabulated (Table 2). It could be observed that the sensitivity ranged from 71% to 94% and 77% to 95%, the precision ranged from 78% to 96% and 94% to 96%, and accuracy ranged from 80% to 88% and 77% to 95% during controlled environment and natural environment trials, respectively (Table 2).

**Table 2:** The average of sensitivity, precision, and accuracy values from Leave-One-Subject-Out Cross-Validation using KNN model for *k* in the range of 3 to 21 for controlled environment and natural environment trials.

	Controlled Environment Trial			Natural Environment Trial		
K	Sensitivity (%)	Precision (%)	Accuracy (%)	Sensitivity (%)	Precision (%)	Accuracy (%)
3	87	86	80	95	94	95
5	83	78	82	92	94	92
7	88	91	83	90	94	90
9	94	93	88	86	94	86
11	87	90	87	85	94	85
13	82	95	87	92	95	92
15	76	94	87	84	95	84
17	75	96	88	82	95	82
19	75	96	88	81	95	81
21	71	95	85	77	96	77
Avg.	82	91	85	86	95	86

## Discussion

Previous studies investigated the ability of different feature extraction and classification methods in recognizing the hand motion pattern for human-machine interface applications and rehabilitation purposes using IMUs, EMG sensors and flex/stretchable sensors [9–16]. Those studies showed that the EMG and flex/stretchable sensors are able to recognize hand and finger motion with high accuracy, sensitivity, and specificity. Additionally, there are studies that specifically investigated the ability of wearable systems to recognize hand-to-face motion [27–34]. Studies that investigated face touch detection were tabulated and compared to our proposed method in Table 3.

**Table 3:** Summary of the proposed algorithms, experimental procedure involved, and obtained outcomes in the studies investigated face touch monitoring.

Author	Highlights	Outcome
Marullo et al. [27]	Proposed a RNN model based on accelerometer readout to detect face touch. In addition to face touch gestures, confounding and common gestures were included in the dataset for evaluation. Model was evaluated based on the data collected from 12 participants.	The true positive rate and false positive rate were 100% and 3.1%, respectively.
Rojas et al. [28]	Proposed a binary classifier model trained based on earphone readout to detect the face touch. In addition to face touch gestures, confounding and common gestures were included in the dataset for evaluation. Model was evaluated based on the data collected from 29 participants.	Sensitivity and precision ranged from 90.0% to 96.0% and 86.5% to 98.3% were obtained, respectively.
D’Aurizio et al. [29]	**Method 1**, Detection with Magnetometer: Proposed an algorithm relied on a smartwatch placed on the hand, a processing unit, and a magnetic wearable accessory (like a necklace) that should be worn close to the face. Accelerometer, gyroscope, and magnetometer readouts were used to detect face touch. The algorithm was evaluated based on the data collected from 10 participants.	Correctly detected rate and false positive rate of 91.3% and 3.2% were obtained.
	**Method 2**, Detection without Magnetometer: Proposed an algorithm relied on the accelerometer and gyroscope readouts obtained by the smartwatch to detect face touch. The algorithm was evaluated based on the data collected from 10 participants.	Correctly detected rate and false positive rate of 92.6% and 38.1% were obtained.
Chen et al. [30]	Investigated a DTW based classifier to detect face touch using smart-watch.	Overall accuracy of 97% and 85% in user-dependent and user-independent tests.
Michelin et al. [31]	Proposed a Convolutional Neural Networks (CNN) model based on the IMU readouts to detect face touch. Their experimental setup was composed of an IMU, a processing unit (laptop), and a cable to connect the IMU to the laptop. The algorithm was evaluated based on the data collected from 40 participants during sitting, standing, and walking trials.	Overall accuracy of 95.7% was obtained.
Alesmaeil et al. [32]	Investigated the ability of IMU and CNN to detect face touch. The model was evaluated based on the data collected from 5 participants.	Overall accuracy of 97% was obtained.
Roy et al. [34]	Proposed a Random Forest algorithm based on the IMU data to detect face touch. In addition to the face touch gestures, confounding gestures were included in the dataset for evaluation. The algorithm was evaluated based on the data collected from 10 participants.	Overall accuracy of 88.4% and Leave-One-Out accuracy of 70.3% were obtained.
Fathian et al. (Our proposed method)	Proposed a KNN model based on the IMU readouts. Features were extracted from the IMU using DTW technique. The algorithm was evaluated based on the data collected from 11 participants in controlled and natural environment trials. In addition to face touch gestures, confounding gestures and common movements were included in the dataset for the evaluation.	Leave-One-Subject-Out Cross-Validation accuracy ranged from 80% to 88% and 77% to 95% during controlled environment and natural environment trials.

The feasibility of using several methods in detecting and monitoring face touch was investigated in the mentioned studies [27–34]. These studies validated their developed methods using different metrics in the context of their experimental procedure. Yet, the validity of the DTW-based algorithm in detecting face touch and distinguishing it from confounding gestures in controlled and natural environment trials was not investigated. In our study, the experimental procedure and the IMU sensor selection were arranged in a way that they had minimal effect on the participants’ pattern of motion. Furthermore, participants were asked to follow their daily life activities during the natural environment trials to ensure that the validity of our proposed method in detecting face touch could be assessed in a setting similar to real life.

The sensitivity, precision, and accuracy obtained with the DTW-KNN method for detecting the hand gesture and distinguishing the face touch from the confounding gestures in our present study were in the same range with the ones previously obtained for face touch and other type of hand motion. Our obtained sensitivity, precision, and accuracy agreed with the results presented in studies that investigated the validity of face touch detection using wearable sensors [27–34] and in the one that classified smoking gesture using the recordings of a wrist-worn IMU [16]. Notably, the wrist-worn IMU in our study showed high accuracy in detecting face touch and distinguish it from several confounding gestures performed by eleven participants in our experimental procedure. This can indicate the generalization of our reported results for real-world daily life. As expected, the detection of face touch was more challenging in natural environment trials compared to controlled environment trials when the dataset was divided into 70% train and 30% test sets. The KNN classifier predicted the face touch with high accuracy in the controlled environment trials regardless of the number of clusters (*k*) as the sensitivity, precision, accuracy, FPR, and FDR did not considerably change with *k*. In natural environment trials, the KNN classifier was able to detect the face touch with substantial sensitivity, precision, and accuracy for *k* ranging from 3 to 21. Yet, unlike controlled environment trials, the sensitivity and accuracy decreased by increasing the value of *k*, caused by an increase in FN and FP. This agrees with the fact that as *k* increases the neighbourhood becomes less distinct and the classifier becomes less sensitive (to decrease the sensitivity the average of the three feature vectors based on acceleration and the average of the three angular velocities were added to the feature space). Similarly, the sensitivity, precision, and accuracy tended to be higher for k<13 during both controlled environment and natural environment trials. Based on our observations, we recommend either *k* = 3 or *k* = 5 for face touch detection in the controlled environment and natural environment trials. The average sensitivity, precision, and accuracy values obtained for different *k* in Leave-One-Subject-Out Cross Validation were higher during the natural environment trials compared to the controlled environment trials. That could be due to the fact that confounding gestures occurred more in the controlled environment trials compared to the natural environment trials that could increase the chance of mislabeling of the face touches. The limited information about the task performed during the natural environment prevent us from any further investigation.

### Limitation and future work

There are a couple of limitations to this study that should be mentioned. First, our proposed method was developed based on data collected from the dominant hand of the participants. Second, although it is very common to use the feedback mechanism to improve personal behaviours like activity level, the effect of feedback mechanisms on behaviour change is not fully discovered. Future works should investigate the effectiveness of feedback that alert a person in lessening the number of face touches. Third, a larger sample size and a more complex experimental protocol should be conducted to account for more variation of face touching to improve the developed data-driven models and assess the efficacy of our proposed DTW-KNN method. Fourth, due to the fact that the DTW score calculation is computationally demanding, in future studies, the feasibility of using our proposed method in real-time detection of face touch should be investigated. Fifth, the train data set was unbalanced and might result in misclassification. In future work, undersampling and oversampling method could be used to balance the training set. Sixth, because the exact time of the face touch was not recorded in the log sheet, the labelling procedure implemented for the natural environment trials may introduce errors that should be considered when discussing the results.

## Conclusion

In this study, we proposed a system to detect face touch and distinguish it from other confounding hand motions using the recordings of a wrist-worn IMU and based on a DTW-KNN model. We investigated, (1) the practicality of using KNN-DTW method in detecting face touch, (2) the validity of our method in daily life circumstances, (3) the feasibility of distinguishing the face touch from other confounding gestures including eating, drinking, scratching head and reaching and picking an object is investigated. To do that, the experimental procedure and the sensor were selected in a way to have minimal effect on the participants’ pattern of motion. Additionally, the data was collected using a stand-alone sensor (that feels like a smartwatch on the participants’ wrist) while the participants were busy with their daily routines without any interruption. Our proposed method was able to detect face touches and distinguish them from confounding motions such as drinking, eating, scratching the head, and reaching and picking an object. The highest sensitivity, precision and accuracy were obtained by the DTW-KNN model with, either 3 or 5 clusters. Our proposed wearable technology can be used for ambulatory monitoring of the hand motion and sending alarm on face touching as a high-risk habit during daily life.
